# Determination of Chlorpromazine, Haloperidol, Levomepromazine, Olanzapine, Risperidone, and Sulpiride in Human Plasma by Liquid Chromatography/Tandem Mass Spectrometry (LC-MS/MS)

**DOI:** 10.1155/2018/5807218

**Published:** 2018-09-02

**Authors:** Abderrezak Khelfi, Mohammed Azzouz, Rania Abtroun, Mohammed Reggabi, Berkahoum Alamir

**Affiliations:** ^1^Department of Toxicology, Bab-El-Oued hospital, Avenue Mohamed Lamine Debaghine, 16009, Algiers, Algeria; ^2^National Center of Toxicology, Avenue Petit Staouali Delly Brahim, 16062, Algiers, Algeria; ^3^Department of Biology and Toxicology, Ait-Idir hospital, Avenue Abderrezak Hahad Casbah, 16017, Algiers, Algeria

## Abstract

**Background and Objective:**

In this study, turbo-ion spray as an interface of tandem mass spectrometry (MS/MS) was performed for sensitive and accurate quantification of chlorpromazine, haloperidol, levomepromazine, olanzapine, risperidone, and sulpiride in plasma samples.

**Methods:**

Separation was performed by gradient reversed phase high-performance liquid chromatography using a mobile phase containing ammonium formiate 2 mM, pH 2.7, and acetonitrile flowing through a Restek PFP Propyl C18 analytical column (50 mm×2.1 mm i.d.) with particle size of 5 *µ*m, at a flow rate of 800 *µ*L/min. Positive ion fragments were detected in multiple reaction monitoring (MRM) mode. Sample preparation was achieved by solid phase extraction (SPE) (Oasis HLB).

**Results:**

Mean extraction recoveries ranged from 82.75% to 100.96%. The standard calibration curves showed an excellent linearity, covering subtherapeutic, therapeutic, and toxic ranges. Intraday and interday validation using quality control (QC) samples were performed. The inaccuracy and imprecision were below 12% at all concentration levels. The limits of detection (LOD) and quantification (LOQ) for all analytes were under therapeutic ranges for all tested analytes. Thus, the proposed method was sensitive enough for the detection and determination of subtherapeutic levels of these antipsychotics in plasma samples. No interference of endogenous or exogenous molecules was observed and no carryover effects were recorded.

**Conclusion:**

According to the results, the proposed method is simple, specific, linear, accurate, and precise and can be applied for antipsychotic analysis in clinical routine. This method was applied for the determination of the tested antipsychotics in plasma samples taken from 71 individuals.

## 1. Introduction

Antipsychotics, also named neuroleptics, are widely used to treat several mental disorders. These drugs are most often prescribed for schizophrenia, hallucinations, mania, sleeping disorders, dementia, and bipolar disorders [1]. Finding the right therapy in such pathologies is difficult and complex therapeutic schemes are common. Therefore, therapeutic drug monitoring (TDM) of antipsychotics can aid in optimizing therapy, nonresponse, pharmacokinetic interactions, or noncompliance [1, 2]. Many antipsychotic drugs have high pharmacokinetic variability and small therapeutic range, so the antipsychotics are administered at relatively low daily dosages. As defined by the “The AGNP-TDM Expert Group Consensus Guidelines: Therapeutic Drug Monitoring in Psychiatry”, therapeutic ranges are narrow and low plasma concentrations are often seen with antipsychotics. Sensitive and specific analytical methods are required for their reliable, accurate, and precise quantification [3].

All antipsychotics may cause unpleasant side effects and severe poisoning after overdose. Suicide and suicide attempts are also frequent in populations using antipsychotics [4] and several intoxications have been published [5, 6]. In order to contribute to optimal drug therapy, a well-organised TDM service with fast turn-around times is very important.

Apart from the target drugs, plasma samples contain numerous endogenous compounds (proteins, acids, bases, and salts). Therefore, preparation of plasma samples prior to analysis is essential to concentrate the drugs (at trace levels) and to remove the proteins and other macromolecules from the matrix. Simple, fast, and universal sample preparation procedure is advantageous, particularly if suitable for different analysis methods. The endogenous compounds could impair the performance of the analytical column, increase the column backpressure, and suppress or intensify the signals during electrospray ionization (ESI) LC-MS/MS analysis. Sample preparation for antipsychotic analyses was mostly performed by liquid-liquid extraction (LLE) [7–13] and SPE [14–17].

At present, determination of some of these drugs is established by high-performance liquid chromatography with UV detection [8, 17–19], coulometric detection [20, 21], and fluorescence detection [7]. Also there are some reports on gas chromatography-mass spectrometry (GC-MS) methods [17] for the determination of antipsychotic drugs, which, however, require derivatization steps. Capillary electrophoresis methods were reported to detect the antipsychotic drugs, but they are not sensitive and robust enough for biological samples [22].

The usefulness of LC-ESI-MS/MS has been demonstrated for a wide range of applications in the bioanalytical filed. Several LC-MS/MS methods have been reported for the quantification of antipsychotic drugs in biological fluids [10, 12, 23–32]. Different sources of ionization have been used (ESI and APCI) although the ESI seems most interesting for the antipsychotic determination in biological samples. All of the analytes were detected in positive ion mode using MRM. The main analyzer used in these works was triple quadrupole.

Considering limited sample volumes, multianalyte procedures for screening and quantification of analytes using mass spectrometry in different biological matrices have become more and more popular in the field of TDM as well as in clinical and forensic toxicology. Multianalyte procedures are also preferable because they make the analytical process much simpler, faster, and cheaper. For unambiguous identification, it is recommended to monitor two or more ion transitions per compound in combination with acceptable tolerance ratios for these transitions.

One of the most important problems when using ESI is the possible reduction or increase of analyte ionization by coeluting compounds. Ionization influence results from the presence of compounds that can change the efficiency of droplet formation or droplet evaporation, which in turn affects the amount of charged ions in the gas phase that ultimately reach the detector. Such effects (suppression or enhancement of ionization) possibly influence the sensitivity, linearity, accuracy, and precision of the assay in quantitative LC-ESI-MS. Sample preparation could reduce (clean-up) or enhance (preconcentrate) matrix effects. Bioanalytical procedures using LC-ESI-MS should only be used routinely and only be accepted if ion suppression studies by sample preparation and/or chromatographic condition optimization have been performed.

The aim of this study is to develop a high throughput LC-MS/MS method for simultaneous identification and quantification of the most commonly used antipsychotics in human plasma. The focus is also on drugs that often occur in poisonings cases. The quantification procedure was fully validated and proved to be suitable for TDM and clinical toxicology. During method development and validation, recovery, matrix effect, linearity, accuracy, precision, LOD, LOQ, selectivity, specificity, carryover, and stability were tested.

## 2. Materials and Methods

### 2.1. Apparatus

An API BioSystem 3200 tandem mass spectrometer, equipped with turbo-ion spray interface was used for measurements. The HPLC system consisted of Perkin-Elmer 200 series autosampler and binary pump, Restek PFP Propyl precolumn (10 mm×2.1 mm) and C18 analytical column (50 mm × 2.1 mm i.d.) with particle size of 5 *µ*m. The mobile phase was degassed using vacuum degasser (Perkin-Elmer 200 series). Data acquisition and processing were achieved using Analyst 1.6 software (Applied Bio-systems).

### 2.2. Reagents

Chlorpromazine, haloperidol, levomepromazine, olanzapine, risperidone, sulpiride, and repaglinide were obtained from the National Laboratory of Pharmaceutical Products of Algiers. HPLC grade acetonitrile and methanol were obtained from Panreac and Sigma-Aldrich, respectively. Ammonium formate and formic acid used as buffer system were obtained from Analar Normapur and Panreca, respectively.

High-purity water for preparative purpose was produced by double distillation for ultrapure deionised water (18.2 MΩ-cm, type I) by bidistillation apparatus (PureLab Option-Q).

### 2.3. Sample and Calibration Standard Preparation

The stock solution was prepared by dissolving 10 mg of chlorpromazine, haloperidol, levomepromazine, olanzapine, risperidone, and sulpiride in methanol to obtain a final concentration of 100 mg/L, which was kept at 4°C until analysis. Work solutions were freshly prepared by an adequate dilution of the stock solution with methanol. The calibration standards were obtained by diluting (1: 20) the corresponding work solution with free blank plasma.

### 2.4. Sample Preparation

In this study, the sample preparation was successfully used to measure antipsychotic concentrations in human plasma samples. Blood samples were collected from patients. After centrifugation at 5,000 rpm for 5 minutes, the obtained plasma samples were transferred to cleaned tubes and kept at 4°C until analysis. 20 *μ*L of the internal standard (repaglinide 1,000 ng/ml) were added to 500 *µ*l of calibration standards, plasma samples, and QC.

Sample preparation consisted of SPE with Oasis HLB cartridges. This extraction includes the following steps:

1. Conditioning of the cartridge with 1 ml of methanol
2. Equilibration with 1ml of distilled water
3. Plasma loading
4. Rinsing with 1ml distilled water solution containing 5% methanol
5. Elution with 1 ml of methanol.

### 2.5. Chromatographic Conditions

An aliquot of 20 *µ*l of each sample and calibration standard was loaded on the column. Gradient reversed phase high-performance liquid chromatography was performed by mobile phase consisting of 90% solvent A (water ammonium formiate 2 mM; pH 2.7) + 10% solvent B (acetonitrile) for 3 minutes and subsequently decreased linearly to 10% solvent A over 4 minutes. The system was reequilibrated to the initial condition over 2 minutes. The reequilibrated condition remained for 1 minute. For all separation process, the mobile phase was set at a flow rate of 800 *µ*L/min.

### 2.6. MS-MS Conditions

The spectrometric measurements were made in positive mode and operated using MRM mode. The optimal instrumental settings are given in Table 1. All given values (compounds and source/gas parameters) are the averages of three measurements (Tables 1 and 2).

### 2.7. Validation Experiments

The proposed method was validated for recovery, matrix effect, LOD, LOQ, selectivity, specificity, carryover, linearity, and stability. In addition, an intra- and interday validation were performed to evaluate the accuracy and precision of the measurements. All these validation experiments were carried out to allow a bioanalytical application of the present method.

## 3. Results and Discussion

### 3.1. Extraction Experiment

In this study, the extraction efficiency (recovery) was evaluated by comparing detector signals (peak areas) obtained from extracts of QC samples at low, medium, and high levels of all tested antipsychotics with those obtained with the corresponding standard solutions added to extracted matrices (Table 3). For all tested antipsychotics, the mean recoveries were more than 80% showing the higher efficiency of the proposed SPE procedure for some analytes compared to those obtained with other methods in previous studies (Table 6). To our knowledge, this is the first work that uses SPE-HLB cartridges for antipsychotic extraction. Although this work uses a higher amount of plasma compared to other methods, the obtained extract was clear and prevented rapid clogging of injection and ESI needles often encountered with simple LLE.

### 3.2. Chromatogram

**Figure 1:**
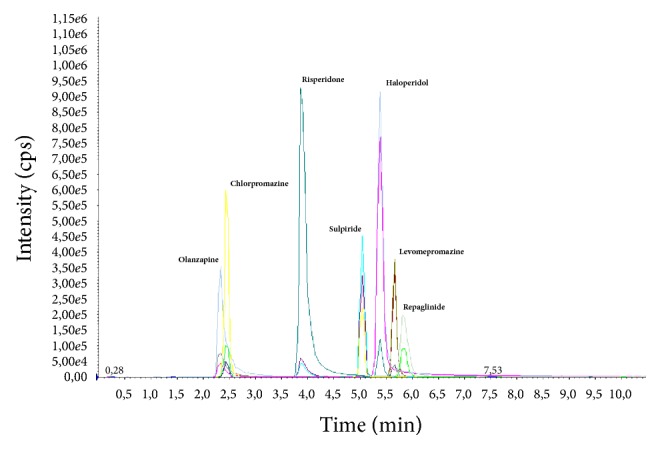
MRM chromatogram of blank plasma sample spiked with antipsychotics (chlorpromazine, haloperidol, levomepromazine, olanzapine, risperidone, and sulpiride) at concentration of 75, 40, 100, 100, 100, and 80 ng/ml, respectively.

**Figure 2:**
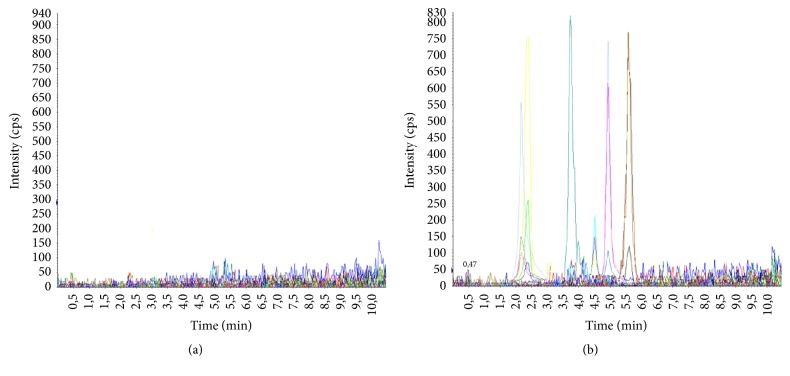
MRM chromatogram of free blank plasma (a) and blank plasma sample spiked with antipsychotics at LOQ (b) (chlorpromazine (1.70 ng/ml), haloperidol (0.49 ng/ml), levomepromazine (2.26 ng/ml), olanzapine (1.01 ng/ml), risperidone (0.52 ng/ml), and sulpiride (1.82 ng/ml)).

Chromatograms with MRM profiles obtained from human plasma containing the tested antipsychotics are shown in Figure 1. Distinct peaks appeared for all compounds with different retention times (Table 4). The total chromatographic run time for analyte separation was 10 minutes, which is suitable for routine analysis as reported in previous works [23, 27, 31]. Representative chromatogram of drug free blank plasma is shown in Figure 2(a).

### 3.3. Linearity

The calibration curve was established with six points of standard solutions with all tested antipsychotics. Each point was determined by five calibration runs. The first-order regression equations with correlation coefficients are shown in Table 4. The linearity of the calibration curves was evaluated by the lack of fit test at 5% level of significance. According to F_reg_ values, the regression explains the observed variations. The F_nl_ values indicate nonsignificant lack of fit of the calibration curve. Therefore, the regression equations established a linear relationship between antipsychotic concentrations and detector signals in the tested ranges. Thus, subtherapeutic, therapeutic, and toxic levels of all tested psychotics can easily be determined without constantly needing of dilution procedures (Table 6).

### 3.4. Accuracy and Precision

In this study, precision and accuracy were determined by intraday and interday validation using QC at four levels (LOQ, 3 LOQ, 50% and 75% of the calibration curve). The intraday validation was performed by five replicate analysis of QC on the same day. For interday validation, five replicate measurements on three different days were performed. Analyte concentrations in QC were calculated using the regression equation of the calibration curve. Accuracy and precision of the analytical method were calculated and the expressed values are summarized in Table 5.

All the coefficients of variation (CV%) of intraday and interday measurements were not greater than 9% and 11%, respectively. These results indicate a multilevel high precision of the present method regardless of time factor. All accuracy measures of intraday and interday validation were less than 10% and 12% (absolute values), respectively, which indicate a multilevel high accuracy of the present method regardless of time factor. Moreover, the intraday precision in all calibration range (repeatability) was acceptable at 5% level of significance according to Cochran test (C (5%)=0.727). The interday precision (reproducibility) in all calibration range was also acceptable at 5% level of significance according to Grubbs test (G (5%)=1.115).

### 3.5. Limits of Detection and Quantification

The LOD and LOQ were calculated using S/N (S/N ≥ 3 for LOD and S/N ≥ 10 for LOQ (Figure 2(b))) as well as the slope and the standard deviation of calibration curve intercepts (3 s(b0)/b and 10 s(b0)/b values, respectively) (Table 4). The highest values between the two approaches were considered as LOD and LOQ for routine analyses. In general, the values of LOD and LOQ using S/N approach were below of those obtained in previous studies and demonstrate that concentrations below therapeutic ranges can be reached and determined (Tables 4 and 6).

### 3.6. Matrix Effect, Selectivity, Specificity, and Carryover

With respect to matrix effect and selectivity, no major interferences (0.2 times the response of the LOQ) were detected at the retention times of all tested antipsychotics or the internal standard in 10 batches of free blank plasma and no suppression effect was found to all tested antipsychotics. In addition, common drugs, when injected into the mass spectrometer, do not generate any interfering ions with those selected for antipsychotic quantification.

Due to the large range of the calibration curve in this method, the carryover was assessed by measuring detector signals (peak areas) of blank samples after the higher calibration point. The accepted limit for carry over was that the detector signals of blank samples must be less than 20% of the LOQ signal. The obtained carryover in this method complied with the acceptable limits.

### 3.7. Stability

The stability of the tested antipsychotics in plasma matrix was estimated using QC stored in different conditions (Table 7). The stability was expressed by the relative bias of the found concentrations to the nominal concentrations. The tested antipsychotics were considered stable when less than 15% of the nominal concentration is obtained using relative bias.

The results indicate that the signs of deterioration of all tested antipsychotics were within the acceptance limits in different conditions (Table 7).

### 3.8. Application

The present method was successfully used in our laboratory for routine analysis of plasma samples taken from patients receiving these antipsychotics in TDM context. The method was also used for clinical analyses of plasma samples obtained from intoxicated individuals. Overall, plasma samples from 71 patients (48 males and 23 females) were analyzed and some of the patients were examined repeatedly. A summary of the obtained results is given in Table 8. Three samples which gave results above the calibration range were diluted with calf serum and reanalyzed.

**Figure 3:**
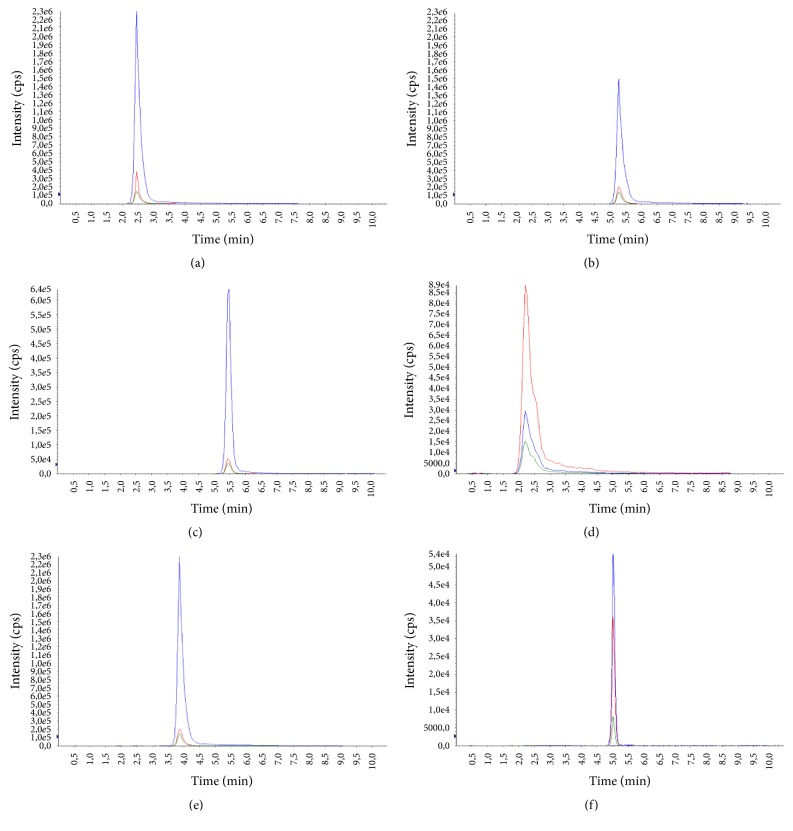
Representative chromatograms of patients' plasma samples containing antipsychotics. (a) Chlorpromazine (322.52 ng/ml), (b) haloperidol (7.31 ng/ml), (c) levomepromazine (161. 83 ng/ml), (d) olanzapine (28.58 ng/ml), (e) risperidone (280.93 ng/ml), and (f) sulpiride (10.37 ng/ml).

The average age of the patients was 29.3 ± 7.4 years (14-69) for males and 37.1 ± 6.9 years (16-72) for females. Examples of representative MRM chromatograms of plasma samples from six different patients taking one of the studied drugs are shown in Figures 3(a)–3(f).

According to our experience, TDM could help to enhance the therapeutic response, design optimal dosing regimens, and avoid the build-up of excessively high and potentially toxic drug concentrations, as well as monitor patient's adherence to treatment.

## 4. Conclusion

A simple method was developed for antipsychotic determination in plasma samples by tandem mass spectrometry detection using turbo-ion spray as an interface. SPE procedure with good recovery was performed in order to render this analytical method relevant in routine clinical diagnosis. For separation, a gradient reversed phase high-performance liquid chromatography with low time consumption was performed.

Validation experiments showed good results in terms of accuracy, precision, and sensitivity in all concentration levels. From the viewpoints of calibration range, this analytical method seems recommendable for TDM and toxicological diagnosis without constantly needing of dilution procedures. The LOD and LOQ appear to be sufficiently low to evaluate subtherapeutic concentrations, which is very useful in therapeutic monitoring.

As an application, this method was applied for antipsychotic determination in 71 plasma samples collected from different cases.

## Figures and Tables

**Table 1:** Optimal instrumental settings.

**Injection**	
Injection volume	20 *μ*l

Injection temperature	40°C

Flush volume	250 *μ*l

Pre-inject Flush	2

Post-inject Flush	2

Flush speed	Medium

**Scan**	

Type of scan	MRM: Multiple reaction monitoring

Mode of scan	Positive

**Source/Gas parameter**	

TEM (temperature)	500 K

GS1 (gas source 1)	40 psi

GS2 (gas source 2)	50 psi

CUR (curtain gas)	10 psi

CAD (collision gas)	Medium

IS (ion spray voltage)	5,000 v

IHE (interface heater)	On

**Table 2:** Optimal compound related settings.

**Antipsychotics**	**Q1 (m/z)**	**Q3 (m/z)**	**DP (v)**	**EP (v)**	**CE (v)**	**CXP (v)**
**Chlorpromazine**	319.1	86.1	65	10	32	4
	319.1	214.1	60	15	30	3
	319.1	246.1	57	10	25	4

**Haloperidol**	376.1	123.0	55	15	55	4
	376.1	165.2	60	10	35	3
	376.1	358.2	47	10	35	2

**Levomepromazine**	329.2	58.0	50	10	45	2
	329.2	100.1	70	15	38	3
	329.2	210.2	50	15	30	4

**Olanzapine**	313.2	198.2	45	20	30	3
	313.2	256.0	62	15	35	2
	313.2	169.1	53	20	40	2

**Risperidone**	411.2	191.2	80	20	40	3
	411.2	110.1	55	20	30	2
	411.2	82.2	54	10	38	3

**Sulpiride**	342.2	112.1	72	15	35	5
	342.2	214.1	45	15	45	3
	342.2	98.1	43	10	45	2

**Repaglinide**	453.3	230.2	32	10	43	3
	453.3	174.2	35	15	45	3

**Table 3:** Recovery values at low, medium, and high levels.

**Antipsychotics**	**Concentration levels (ng/ml)**	**Recovery (**%**)**	**Mean recovery (**%**)**
**Chlorpromazine**	15	83.75	82.75
	150	82.69	
	450	81.82	

**Haloperidol**	2	96.22	99.69
	20	100.73	
	60	102.12	

**Levomepromazine**	5	95.24	93.66
	50	94.53	
	150	91.20	

**Olanzapine**	5	77.77	85.23
	50	84.90	
	150	93.01	

**Risperidone**	5	98.75	95.96
	50	94.75	
	150	94.39	

**Sulpiride**	40	100.28	100.96
	400	98.37	
	1,200	104.22	

**Table 4:** Retention times, calibration curve linearity, LOD, and LOQ.

**Antipsychotics**	**Rt(min)**	**Therapeutic range (ng/ml)**	**Concentration range (ng/ml)**	**Regression equations**	**R** ^**2**^	**LOD (ng/ml)**	**LOQ (ng/ml)**
**Chlorpromazine**	2.45±0.04	30-300	15-450	y = 0.06762x +	0.99965	3.95 (0.53)	13.17 (1.70*∗*)
			(15, 30, 75, 150, 300 and 450)	0.17059			

**Haloperidol**	5.38±0.05	5-17	2-60	y = 0.18126x +	0.99902	0.36 (0.11)	1.19 (0.49*∗*)
			(2, 4, 10, 20, 40 and 60)	0.06470			

**Levomepromazine**	5.62±0.08	15-60	5-150	y = 0.03452x +	0.99927	1.50 (0.80)	4.99 (2.26*∗*)
			(5, 10, 25, 50, 100 and 150)	0.01757			

**Olanzapine**	2.34±0.05	20-80	5-150	y = 0.03241x -	0.99915	0.87 (0.33)	2.89 (1.01*∗*)
			(5, 10, 25, 50, 100 and 150)	0.01622			

**Risperidone**	3.88±0.05	20-60	5-150	y = 0.08304x +	0.99975	1.378 (0.15)	4.59 (0.52*∗*)
			(5, 10, 25, 50, 100 and 150)	0.01389			

**Sulpiride**	5.07±0.05	200-1,000	40-1,200	y = 0.04711x +	0.99984	2.11 (0.41)	7.04 (1.82*∗*)
			(40, 80, 200, 400, 800 and 1,200)	0.05818			

(*∗*): LOD and LOQ using S/N approach.

**Table 5:** Precision and accuracy values.

**Antipsychotics**	**Concentration level (ng/ml)**	**Precision (CV**%**)**		**Accuracy ** **(relative ** **bias**%**)**	
		**Intraday assay (n=5)**	**Interday assay** **(n = 5 within 3 days)**	**Intraday assay (n=5)**	**Interday assay** **(n = 5 within 3 days)**
**Chlorpromazine**	15	7.15	1.09	5.97	-4.87
	30	7.75	9.16	9.60	-2.40
	75	4.23	1.65	-1.12	-1.21
	150	4.01	4.69	-3.93	0.48
	300	4.58	1.50	0.53	1.67
	450	4.09	0.63	0.18	-0.74
	CQ1 (13)	4.01	8.50	8.62	6.50
	CQ2 (39)	4.36	1.20	8.67	6.12
	CQ3 (160)	5.15	7.71	0.14	4.80
	CQ4 (250)	3.18	1.91	1.06	2.42

**Haloperidol**	2	8.58	1.10	-9.02	-5.15
	4	2.20	1.25	-3.00	-0.99
	10	3.98	1.35	-4.36	-3.53
	20	1.98	0.61	2.39	1.59
	40	3.63	0.22	2.93	1.65
	60	4.37	1.26	-1.42	-0.80
	CQ1 (1)	3.66	6.54	4.52	9.65
	CQ2 (3)	3.52	8.56	-4.69	-0.13
	CQ3 (30)	1.13	0.62	7.16	6.00
	CQ4 (45)	1.78	0.87	0.35	-0.66

**Levomepromazine**	5	3.35	6.10	0.78	11.92
	10	3.12	6.31	8.77	10.55
	25	2.69	2.85	-1.05	-2.18
	50	6.08	1.95	-5.12	-6.56
	100	6.88	2.86	1.85	2.50
	150	4.82	0.96	-0.27	-0.40
	CQ1 (2)	2.16	7.17	-3.57	11.01
	CQ2 (6)	3.54	9.77	9.73	11.26
	CQ3 (80)	2.41	2.14	7.82	3.01
	CQ4 (120)	3.21	1.43	0.67	-1.90

**Olanzapine**	5	1.56	2.22	-5.66	5.13
	10	5.79	3.47	3.19	10.40
	25	5.51	3.59	-6.41	-0.18
	50	2.80	1.12	-7.91	-5.97
	100	2.72	2.33	0.64	1.82
	150	3.15	0.79	-0.74	-0.22
	CQ1 (3)	1.79	7.25	-5.05	9.78
	CQ2 (9)	3.87	3.31	-8.60	1.54
	CQ3 (80)	5.10	1.68	-0.09	3.14
	CQ4 (120)	4.72	2.52	3.37	-1.13

**Risperidone**	5	7.58	2.78	6.48	2.00
	10	4.28	1.77	-0.88	-6.12
	25	5.01	4.69	-5.73	-4.09
	50	4.70	1.04	2.49	2.52
	100	2.24	0.62	0.42	1.48
	150	2.63	0.56	-0.30	-0.79
	CQ1 (5)	3.58	2.81	4.36	-1.17
	CQ2 (15)	4.36	1.17	4.54	5.04
	CQ3 (80)	1.72	0.23	-1.04	0.41
	CQ4 (120)	1.75	0.57	-2.01	0.05

**Sulpiride**	40	3.99	5.91	9.60	9.10
	80	1.82	1.30	7.34	7.01
	200	5.08	1.37	-4.52	-4.55
	400	3.73	2.01	-1.12	-0.34
	800	2.63	1.22	0.09	-0.10
	1,200	1.17	1.79	0.18	0.17
	CQ1 (7)	4.62	5.15	-7.46	-4.13
	CQ2 (21)	2.67	10.28	-8.20	-1.40
	CQ3 (600)	2.42	2.16	-0.54	0.13
	CQ4 (800)	2.23	0.53	0.15	-1.09

**Table 6:** Recovery, concentration range, LOD, and LOQ from different mass spectrometry methods.

**Antipsychotics**	**Extraction recovery (**%**)**	**Concentration range (ng/ml)**	**LOD (ng/ml)**	**LOQ (ng/ml)**
**Chlorpromazine**	67 [10]	30- 300 [10]	11.3 [24]	15 [24]
	84.5 [28]	15-600 [28]	7.5 [28]	15 [28]
	94.13 [32]	10-1,000 [30]	0.3 [32]	1 [29]
		1-50 [32]		1 [32]

**Haloperidol**	65 [10]	5-17 [10]	3.8 [24]	5 [24]
	93.97 [23]	1-60 [26]	<0.5 [27]	1 [26]
	86.95 [12]	2.5-30 [28]	1 [28]	1 [27]
	88.5 [33]	1-20 [30]	0.3 [32]	2.5 [28]
	83 [28]	1-50 [32]		0.5 [29]
				0.23 [30]
				1 [32]

**Levomepromazine**	70 [10]	15-60 [10]	5 [28]	7.5 [24]
	81 [28]	10-300 [28]		10 [28]
		10-1,000 [30]		0.47 [30]

**Olanzapine**	92.02 [23]	2-200 [25]	1 [27]	2 [25]
	96 [25]	10-160 [28]	2 [28]	5 [27]
	93.95 [12]	10-1,000 [30]		10 [28]
	102 [33]			0.5 [29]
	77 [28]			1.83 [30]
	86 [31]			

**Risperidone**	69 [10]	5- 60 [10]	1.9 [24]	2.5 [24]
	89.6 [23]	1.5-60 [28]	<1 [27]	5 [27]
	94 [25]	1-50 [30]	0.8 [28]	1.5 [28]
	88.85 [12]			0.67 [30]
	84 [33]			
	80.5 [28]			
	88.5 [31]			

**Sulpiride**	12 [10]	200-1,000 [10]	75 [24]	100 [24]
	105 [28]	100-1,500 [28]	2 [27]	20 [27]
		100-10,000 [30]	80 [28]	100 [28]
				8.3 [30]

**Sample volume (** **µ** **l): **50 [10], 200 [12], 250 [23], 500 [24], 200 [25], 100 [26], 500 [27], 500 [28], 200 [29], 100 [30], 250 [31], 200 [32], and 500 [33].

**Sample preparation technique: **LLE [10, 12, 23–26, 28, 30, 31, 33]; SPE [27, 29, 32].

**Table 7:** Evaluation of sample storage procedure.

**Antipsychotics**	**Nominal concentrations of QC (ng/ml)**	**Stability: Storage condition of samples**
Chlorpromazine	13	Four freeze/thaw cycles
	39	Bench top (6 h)
	160	Autosampler stability (24 h)
	250	Preserved during 1 month at -20°C

Haloperidol	1	Three freeze/thaw cycles
	3	Bench top (6 h)
	30	Autosampler stability (12 h)
	45	Preserved during 1 month at -20°C

Levomepromazine	2	Four freeze/thaw cycles
	6	Bench top (6 h)
	80	Autosampler stability (24 h)
	120	Preserved during 1 month at -20°C

Olanzapine	3	Four freeze/thaw cycles
	9	Bench top (6 h)
	80	Autosampler stability (24 h)
	120	Preserved during 1 month at -20°C

Risperidone	5	Three freeze/thaw cycles
	15	Bench top (6 h)
	80	Autosampler stability (24 h)
	120	Preserved during 1 month at -20°C

Sulpiride	7	Four freeze/thaw cycles
	21	Bench top (6 h)
	600	Autosampler stability (24 h)
	800	Preserved during 1 month at -20°C

**Table 8:** Overview of antipsychotic determination in plasma samples taken from 71 patients.

**Antipsychotics**	**Number of positive cases**	**Mean (ng/ml)**	**Minimum (ng/ml)**	**Maximum (ng/ml)**	**<** **therapeutic range**	**>** **therapeutic range**
**Chlorpromazine**	13	270.76	21.44	440.77	3	4

**Haloperidol**	30	27.42	1.29	131.46	4	21

**Levomepromazine**	8	79.10	17.08	375.27	0	4

**Olanzapine**	9	141.33	18.31	168.46	1	3

**Risperidone**	6	116.70	12.34	280.93	1	1

**Sulpiride**	13	800.61	10.37	1431.72	2	7

## Data Availability

All data are provided in full in the results section of this article.

## Additional Points

*Highlights*. This method includes the following highlights: (i) enhanced extraction recoveries; (ii) excellent linearity; (iii) high precision and accuracy; (iv) low limit of detection and quantification for all tested antipsychotics; (v) no matrix effect detected; (vi) application in routine analysis for 71 individuals.
